# A single intranasal dose of a live-attenuated parainfluenza virus-vectored SARS-CoV-2 vaccine is protective in hamsters

**DOI:** 10.1073/pnas.2109744118

**Published:** 2021-12-07

**Authors:** Xueqiao Liu, Cindy Luongo, Yumiko Matsuoka, Hong-Su Park, Celia Santos, Lijuan Yang, Ian N. Moore, Sharmin Afroz, Reed F. Johnson, Bernard A. P. Lafont, Craig Martens, Sonja M. Best, Vincent J. Munster, Jaroslav Hollý, Jonathan W. Yewdell, Cyril Le Nouën, Shirin Munir, Ursula J. Buchholz

**Affiliations:** ^a^RNA Viruses Section, Laboratory of Infectious Diseases, National Institute of Allergy and Infectious Diseases, National Institutes of Health, Bethesda, MD 20892;; ^b^Infectious Disease and Pathogenesis Section, Comparative Medicine Branch, National Institute of Allergy and Infectious Diseases, National Institutes of Health, Bethesda, MD 20892;; ^c^SARS-CoV-2 Virology Core, Laboratory of Viral Diseases, National Institute of Allergy and Infectious Diseases, National Institutes of Health, Bethesda, MD 20892;; ^d^Research Technologies Section, Rocky Mountain Laboratories, National Institute of Allergy and Infectious Diseases, National Institutes of Health, Hamilton, MT 59840;; ^e^Laboratory of Virology, Rocky Mountain Laboratories, National Institute of Allergy and Infectious Diseases, National Institutes of Health, Hamilton, MT 59840;; ^f^Cellular Biology Section, Laboratory of Viral Diseases, National Institute of Allergy and Infectious Diseases, National Institutes of Health, Bethesda, MD 20892

**Keywords:** parainfluenza virus vaccines, COVID-19, SARS-CoV-2, intranasal immunization, vaccine vector

## Abstract

Single-dose vaccines with the ability to restrict SARS-CoV-2 replication in the respiratory tract are needed for all age groups, aiding efforts toward control of COVID-19. We developed a live intranasal vector vaccine for infants and children against COVID-19 based on replication-competent chimeric bovine/human parainfluenza virus type 3 (B/HPIV3) that express the native (S) or prefusion-stabilized (S-2P) SARS-CoV-2 S spike protein, the major protective and neutralization antigen of SARS-CoV-2. B/HPIV3/S and B/HPIV3/S-2P replicated as efficiently as B/HPIV3 in vitro and stably expressed SARS-CoV-2 S. Prefusion stabilization increased S expression by B/HPIV3 in vitro. In hamsters, a single intranasal dose of B/HPIV3/S-2P induced significantly higher titers compared to B/HPIV3/S of serum SARS-CoV-2–neutralizing antibodies (12-fold higher), serum IgA and IgG to SARS-CoV-2 S protein (5-fold and 13-fold), and IgG to the receptor binding domain (10-fold). Antibodies exhibited broad neutralizing activity against SARS-CoV-2 of lineages A, B.1.1.7, and B.1.351. Four weeks after immunization, hamsters were challenged intranasally with 10^4.5^ 50% tissue-culture infectious-dose (TCID_50_) of SARS-CoV-2. In B/HPIV3 empty vector-immunized hamsters, SARS-CoV-2 replicated to mean titers of 10^6.6^ TCID_50_/g in lungs and 10^7^ TCID_50_/g in nasal tissues and induced moderate weight loss. In B/HPIV3/S-immunized hamsters, SARS-CoV-2 challenge virus was reduced 20-fold in nasal tissues and undetectable in lungs. In B/HPIV3/S-2P–immunized hamsters, infectious challenge virus was undetectable in nasal tissues and lungs; B/HPIV3/S and B/HPIV3/S-2P completely protected against weight loss after SARS-CoV-2 challenge. B/HPIV3/S-2P is a promising vaccine candidate to protect infants and young children against HPIV3 and SARS-CoV-2.

## Significance

Pediatric SARS-CoV-2 infections, though generally mild, are associated with substantial morbidity and contribute to transmission dynamics. No SARS-CoV-2 vaccines are available for young children. Bovine/human parainfluenza virus 3 (B/HPIV3) vectors for intranasal immunization of children were evaluated previously in phase 1/2 studies and were well-tolerated in children as young as 2 mo of age. This manuscript describes a B/HPIV3 vector expressing a prefusion-stabilized version of the SARS-CoV-2 S protein (S-2P), and shows that a single intranasal dose is highly immunogenic and protective against SARS-CoV-2 challenge in the hamster model, the most robust SARS-CoV-2 challenge model available. Based on these results, B/HPIV3/S-2P represents a promising vaccine candidate for clinical evaluation as a pediatric vaccine for intranasal immunization against HPIV3 and SARS-CoV-2.

The betacoronavirus severe acute respiratory syndrome coronavirus 2 (SARS-CoV-2) emerged in 2019 and rapidly spread globally (1). In the first year of the pandemic, over 105 million infections and 2.3 million deaths have been reported worldwide, including over 27 million cases and 500,000 deaths in the United States (https://covid19.who.int/). Vaccines are being rapidly deployed in a race to control ongoing infections and the emergence of variants of concern (2), with increased virulence and altered antigenicity.

SARS-CoV-2 infects and spreads primarily via the respiratory route (3, 4), and mucosal surfaces of the respiratory tract represent the primary site of infection. COVID-19, the disease caused by SARS-CoV-2, is characterized by upper and lower respiratory tract symptoms, fever, chills, body aches, and fatigue, and in some cases gastrointestinal and other symptoms with involvement of additional tissues (5, 6).

SARS-CoV-2 infection is initiated by the spike (S) surface glycoprotein, the main target for SARS-CoV-2–neutralizing antibodies. The S protein is a trimeric class I fusion glycoprotein. Each protomer consists of two functionally distinct subunits, S1 and S2, linked by a furin cleavage site; S2 contains an additional proteolytic cleavage site S2′. S2/S2′ cleavage is mediated by the transmembrane protease serine 2 (TMPRSS2) (1, 7–9). The S1 subunit contains the receptor-binding domain (RBD). The S2 subunit contains the membrane fusion machinery, including the hydrophobic fusion peptide and α-helical heptad repeats (7, 9).

Binding of the S RBD to its receptor, human angiotensin converting enzyme 2, triggers a change, from the closed and metastable prefusion conformation to the open and stable postfusion form that drives membrane fusion enabling viral entry (1). Stabilization of the S protein in its native prefusion state should preserve antibody epitopes, including immunodominant sites of the RBD, required to elicit high-quality neutralizing antibody responses (9–13). Thus, a prefusion-stabilized version of the S protein is the optimal vaccine immunogen (13–15).

Vaccines for SARS-CoV-2 are available, but currently are limited to individuals 12 y of age or older. They are administered intramuscularly, which does not directly stimulate mucosal immunity in the respiratory tract, the primary site of SARS-CoV-2 infection and shedding. While the major burden of COVID-19 disease is in adults, infection and disease also occurs in infants and young children, contributing to viral transmission. Therefore, the development of safe and effective pediatric COVID-19 vaccines is critical for worldwide control of COVID-19. The ideal vaccine should be effective at a single dose, inducing durable and broad systemic immunity, as well as T and B cell respiratory mucosal immunity that completely blocks SARS-CoV-2 infection and transmission.

Here we describe a vectored SARS-CoV-2 vaccine candidate for intranasal immunization of infants and young children. The vaccine is based on an attenuated, replication-competent parainfluenza virus type 3 (PIV3) vector called B/HPIV3 (16) expressing the SARS-CoV-2 S protein. B/HPIV3 consists of bovine PIV3 (BPIV3) strain Kansas in which the BPIV3 hemagglutinin-neuraminidase (HN) and fusion (F) glycoproteins (the two PIV3 neutralization antigens) have been replaced by those of human PIV3 strain JS (16, 17). The BPIV3 backbone provides host range restriction of replication in humans, serving as the basis for strong and stable attenuation (17, 18). B/HPIV3 originally was developed as a live vaccine candidate against HPIV3, and was well-tolerated in young children (17). Moreover, B/HPIV3 has been used to express the F glycoprotein of another human respiratory pathogen, human respiratory syncytial virus (HRSV), as a bivalent HPIV3/HRSV vaccine candidate. This vaccine candidate was well-tolerated in children >2 mo of age (18) (Clinicaltrials.gov NCT00686075), and optimized versions are in further clinical development as pediatric vaccines (19, 20). In the present study, we used B/HPIV3 to express wild-type (S) or prefusion-stabilized (S-2P) versions of the SARS-CoV-2 S protein, creating the vaccine candidates B/HPIV3/S and B/HPIV3/S-2P. These were evaluated in vitro and in hamsters as live-attenuated SARS-CoV-2 intranasal vaccine candidates.

## Results

### Design and In Vitro Characterization of B/HPIV3 Expressing Wild-Type or Prefusion-Stabilized Uncleaved SARS-CoV-2 S Protein.

**Fig. 1.**
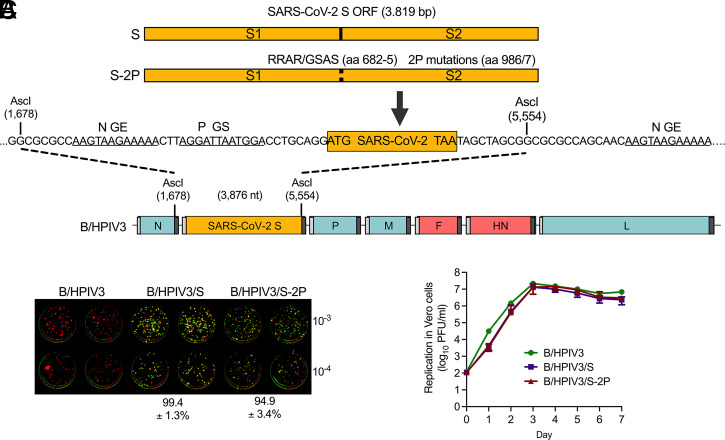
B/HPIV3 vectors expressing wild-type and prefusion-stabilized versions of the SARS-CoV-2 S protein with S1/S2 cleavage site ablated. (*A*) Map of the B/HPIV3 genome with the added SARS-CoV-2 S gene. BPIV3 genes are in blue, HPIV3 genes are in red, and the S gene is in yellow. Each gene, including the SARS-CoV-2 S gene, begins and ends with PIV3 gene start (GS) and gene end (GE) transcription signals (light and dark gray bars, respectively). The S gene encodes either the wild-type (S) or a prefusion-stabilized uncleaved (S-2P) version of the S protein (9), and was inserted into an AscI restriction site to place it between the B/HPIV3 N and P genes. The two stabilizing proline substitutions (amino acids 986/7) and four amino acid substitutions that ablate the furin cleavage site (RRAR to GSAS, amino acids 682 to 685) in the S-2P protein are indicated. (*B*) Stability of SARS-CoV-2 S expression, analyzed by dual-staining immunoplaque assay. Virus stocks were titrated on Vero cells, and analyzed by dual-staining immunoplaque assay essentially as described (20), using a goat hyperimmune antiserum against a recombinantly expressed secreted version of S-2P protein and a rabbit hyperimmune antiserum against HPIV3 virions. HPIV3- and SARS-CoV-2 S-specific staining was pseudocolored in red and green, respectively; dual staining appeared as yellow. The percentage (±SD) of yellow plaques is indicated at the bottom. (*C*) Multicycle replication of B/HPIV3 vectors on Vero cells. Vero cells in six-well plates were infected in triplicate with indicated viruses at an MOI of 0.01 PFU per cell and incubated at 32 °C for a total of 7 d. At 24-h intervals, aliquots of culture medium were collected and flash-frozen for subsequent immunoplaque titration on Vero cells.

B/HPIV3 consists of BPIV3 with F and HN genes replaced by those of HPIV3 (16) (Fig. 1*A*). We codon-optimized the 1,273-aa S ORF derived from the first available SARS-CoV-2 genome sequence (GenBank MN908947) (21) for human expression, and placed it under the control of PIV3 gene start (transcription initiation) and end (transcription termination and polyadenylation) signals to direct its expression as a separate mRNA by the PIV3 transcriptional machinery (Fig. 1*A*). We generated a second version of this gene (S-2P) to contain two prefusion-stabilizing proline substitutions at amino acid positions K986P and V987P of S, as well as four amino acid substitutions in the S1/S2 furin cleavage site (residues 682 to 685; RRAR-to-GSAS) that ablate S1/S2 cleavage (9). We inserted each of the two S gene constructs into full-length B/HPIV3 cDNA between the N and P genes (Fig. 1*A*). In previous studies, this enabled efficient and stable expression of heterologous genes with minimal effect on B/HPIV3 vector replication (22). We used the resultant cDNAs to recover recombinant B/HPIV3/S and B/HPIV3/S-2P viruses by reverse genetics, as described previously (23). We propagated virus stocks in Vero cells, which are used for manufacture of clinical-grade material for human administration. Sequencing of full-length viral genomes confirmed the absence of detectable mutations.

We determined viral titers and evaluated the stability of S and S-2P expression using dual-staining immunoplaque assays with polyclonal hyperimmune antisera, one against PIV3 virions and the other against a recombinantly expressed secreted version of S-2P protein. In stocks grown from four (B/HPIV3/S) or eight (B/HPIV3/S-2P) independent recoveries, staining for both PIV3 and SARS-CoV-2 was obtained for 99.4 ± 1.3 and 94.9 ± 3.4% of plaques, respectively (Fig. 1*B*, yellow), indicative of stable expression of SARS-CoV-2 S protein. Multicycle replication of B/HPIV3/S and B/HPIV3/S-2P in Vero cells was efficient and overall similar to that of B/HPIV3, indicating that the presence of the 3.8-kb S or S-2P insert did not detectably impede replication of the B/HPIV3 vector (Fig. 1*C*).

**Fig. 2.**
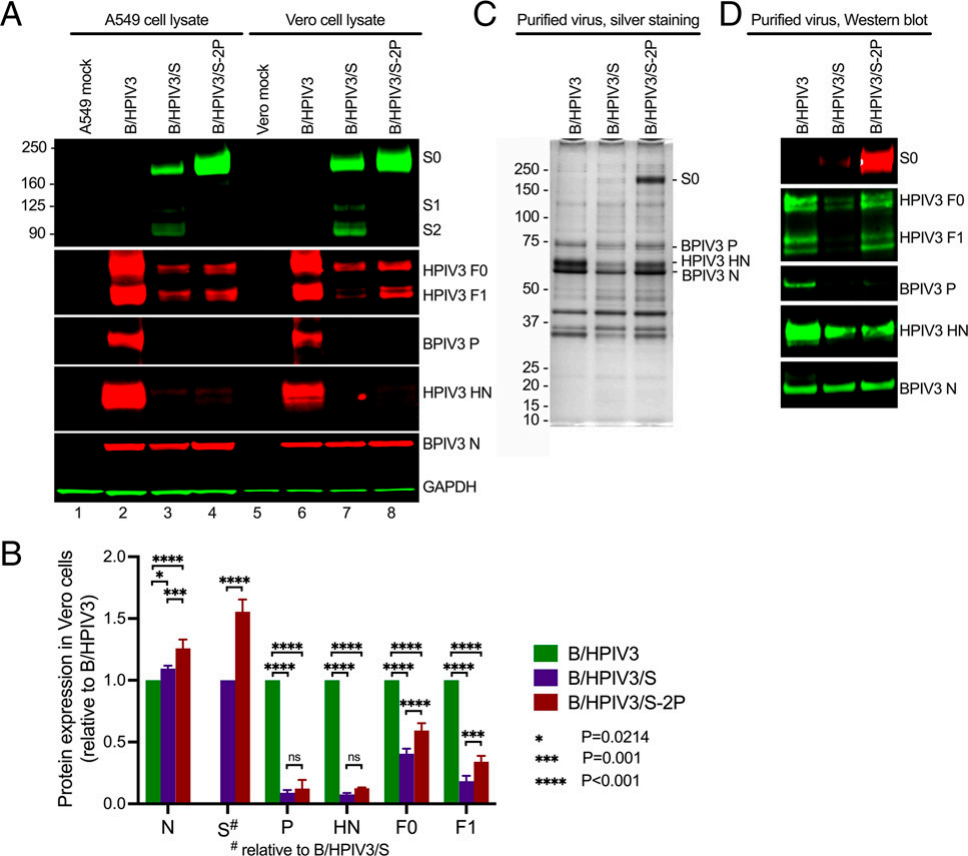
Viral proteins in infected cell lysates and purified virions. (*A*) Vero or A549 cells in six-well plates were infected with B/HPIV3, B/HPIV3/S, and B/HPIV3/S-2P at an MOI of 1 PFU per cell and incubated at 32 °C for 48 h. Cell lysates were prepared, denatured, and analyzed by Western blotting. SARS-CoV-2 S protein was detected by a goat hyperimmune serum to the S protein. The BPIV3 N and P proteins (approximal molecular mass: ∼60 kDa and ∼75 kDa) were detected by a rabbit hyperimmune serum raised against sucrose-purified HPIV3; the HPIV3 HN protein (∼65 kDa) was detected by a rabbit hyperimmune serum against a peptide derived from HPIV3 HN, and the HPIV3 F protein (F_0_: ∼70 kDa; F_1_: ∼48 kDa) was detected by a rabbit hyperimmune serum against the recombinant purified F ectodomain, followed by immunostaining with infrared fluorophore labeled secondary antibodies and infrared imaging. Images were acquired and analyzed using Image Studio software (LiCor). Immunostaining for GAPDH is shown as a loading control. (*B*) Relative expression of N, S, P, HN, and F proteins in Vero cells by B/HPIV3/S and B/HPIV3/S-2P. To obtain these data, three additional, replicate infections were performed. Vero cell lysates were evaluated side-by-side in Western blots as described in *A* (see *SI Appendix*, Fig. S1 for Western blot image). Protein expression was normalized to GAPDH and expressed as fold-change compared to B/HPIV3 in the same experiment. Expression of the SARS-CoV-2 S protein is shown relative to B/HPIV3/S. (*C* and *D*) Silver staining (*C*) and Western blot analysis (*D*) of sucrose-purified B/HPIV3, B/HPIV3/S, and B/HPIV3/S-2P. Vero-grown virus was purified by centrifugation through 30%/60% discontinuous sucrose gradients, and gently pelleted by centrifugation to remove sucrose. One microgram of protein per lane was used for SDS/PAGE, and gels were subjected to silver staining (*C*) and Western blot analysis (*D*).

To characterize the expression of the SARS-CoV-2 S and B/HPIV3 proteins in vitro, we infected Vero and human lung epithelial A549 cells with B/HPIV3 (negative control), B/HPIV3/S, or B/HPIV3/S-2P at a multiplicity of infection (MOI) of 1 plaque-forming unit (PFU) per cell. Cell lysates were prepared 48 h after infection and analyzed by SDS/PAGE (under reducing and denaturing conditions) and Western blotting using the hyperimmune antiserum against the secreted version of SARS-CoV-2 S-2P or PIV3 antigens (Fig. 2*A*). For both recombinant viruses, we detected S protein in lysates as a band consistent in migration with the uncleaved S_0_ precursor protein (Fig. 2*A*, lanes 3, 4, 7, and 8, and *SI Appendix*, Fig. S1). In B/HPIV3/S-infected A549 and Vero cells (Fig. 2*A*, lanes 3 and 7, and *SI Appendix*, Fig. S1), additional smaller products were present, consistent in size with the cleavage products S1 and S2. The absence of these smaller bands in B/HPIV3/S-2P–infected A549 and Vero cells confirmed the absence of proteolytic cleavage of the S-2P protein in which the furin cleavage site had been ablated (Fig. 2*A*, lanes 4 and 8, and *SI Appendix*, Fig. S1). Notably, prefusion stabilization increased the intensity of Western blot staining for the S protein band in both cell lines (Fig. 2*A*, compare B/HPIV3/S and B/HPIV3/S-2P, lanes 3 vs. 4 and 7 vs. 8; and *SI Appendix*, Fig. S1).

Quantitative comparison in Vero cells of protein expression by B/HPIV3/S and B/HPIV3/S-2P from three independent experiments showed that prefusion stabilization increased the levels of vector-expressed SARS-CoV-2 S protein by about 1.6-fold (Fig. 2*B* and *SI Appendix*, Fig. S1*A*). We also investigated how the insertion of an S gene cassette between the BPIV3 N and P genes affected the expression of the backbone vector genes. The quantitative analysis revealed that the level of expression of the upstream N gene by B/HPIV3/S and B/HPIV3/S-2P was slightly increased compared to that of B/HPIV3, while the expression of downstream vector genes (BPIV3 P; HPIV3 F and HN) was reduced by 40 to 90% (Fig. 2 *A* and *B* and *SI Appendix*, Fig. S1).

To evaluate possible incorporation of the SARS-CoV-2 S or S-2P protein into B/HPIV3 particles, we purified Vero cell-grown viruses by ultracentrifugation through sucrose gradients and analyzed the protein composition by gel electrophoresis with silver staining and Western blotting (Fig. 2 *C* and *D*). In silver-stained gels, a high-molecular band consistent with SARS-CoV-2 S0 was visible in B/HPIV3/S-2P preparations, but not in B/HPIV3 or B/HPIV3/S preparations. Immunostaining identified this band as S_0_, and also revealed the presence of a small amount of S_0_ in B/HPIV3/S preparations (Fig. 2*D*), indicating that the prefusion-stabilized version of the S protein was more abundantly incorporated in the B/HPIV3 vector particles than the wild-type version of the S protein.

### Immunization of Hamsters with the B/HPIV3/S and B/HPIV3/S-2P.

**Fig. 3.**
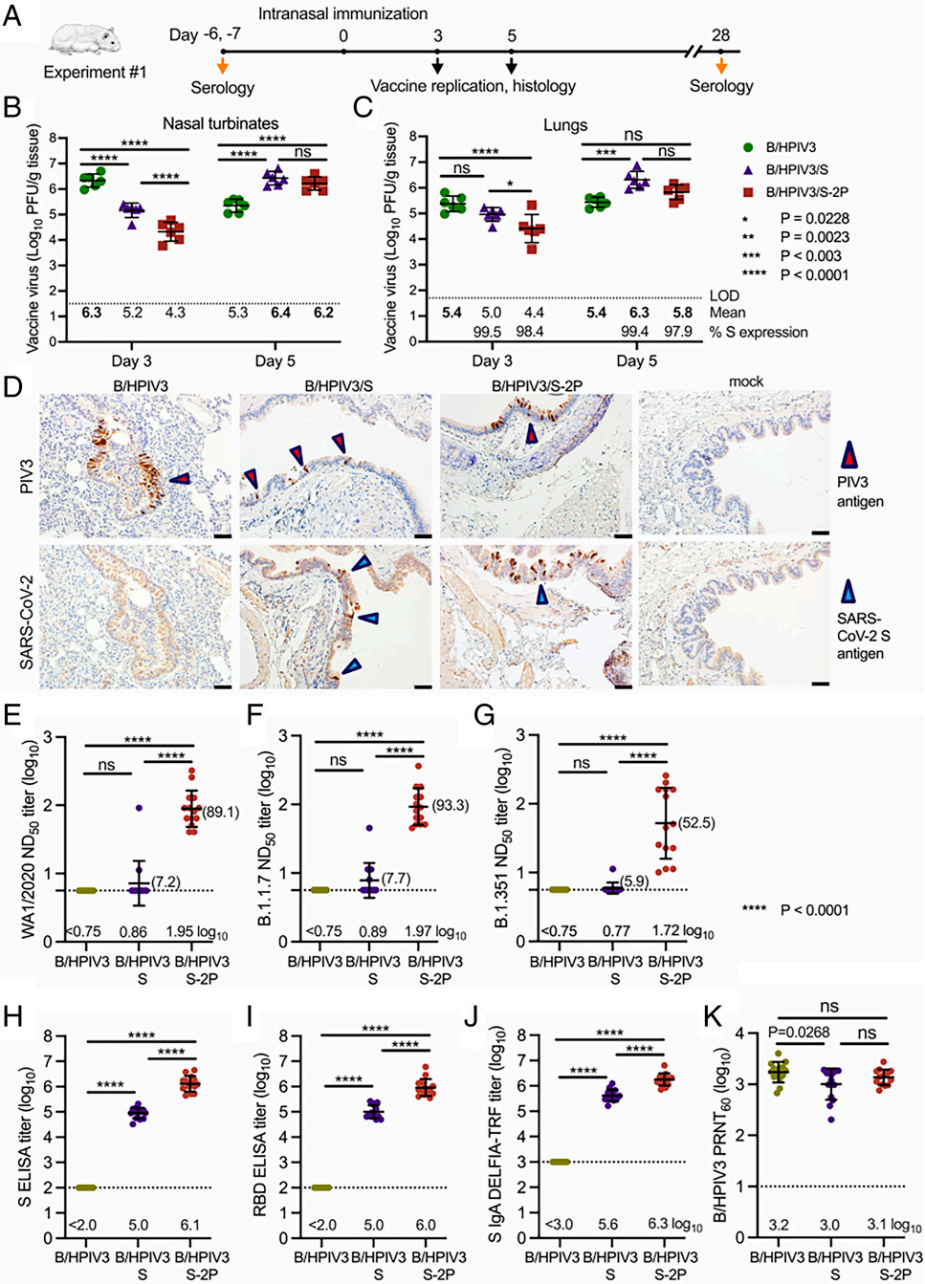
Replication and immunogenicity in the hamster model. (*A*) In Exp. 1, 6-wk-old golden Syrian hamsters in groups of 30 were inoculated intranasally with 5 log_10_ PFU of the indicated viruses. On days 3 and 5, six animals per group per day were killed and the viral titers in the nasal turbinates (*B*) and lungs (*C*) were determined by dual-staining immunoplaque assay. Individual animal titers are shown by symbols and group means are shown immediately below the dotted line; the maximum mean peak titer irrespective of day for each group is in bold. The limit of detection (LOD) was 50 PFU/g of tissue. In *C*, the average percentage of dual-stained plaques is indicated immediately above the *x* axis, indicating stability of S expression of the B/HPIV3 vectors during in-vivo replication. (*D*) On days 3 and 5, lung tissues were obtained (*n* = 2 animals per group) and processed for IHC analysis. Serial sections were immunostained for HPIV3 and SARS-CoV-2 antigen using hyperimmune antisera against HPIV3 virions and secreted S-2P protein, respectively. Representative images from day 5 are shown. Areas with bronchial epithelial cells positive for HPIV3 and SARS-CoV-S are marked by red and blue arrowheads, respectively (20× magnification). (Scale bar, 50 µm.) (*E*–*K*) Sera were collected on day 28 and serum antibody titers were evaluated (*n* = 14 animals per group) to determine the 50% SARS-CoV-2 neutralizing titers (ND_50_) on Vero E6 cells against isolates WA1/2020 (lineage A), USA/CA_CDC_5574/2020 (lineage B.1.1.7), and USA/MD-HP01542/2021 (lineage B.1.351) (*E*–*G*), or IgG ELISA titers to a secreted form of the S-2P protein (*H*) or to a fragment of the S protein (amino acids 328 to 531) containing SARS-CoV-2 RBD (*I*), or IgA titers to a secreted form of the S-2P protein, determined by DELFIA-TRF (*J*). (*K*) The sera also were analyzed to determine 60% PRNT_60_ to B/HPIV3. Mean log_10_ antibody titers are indicated immediately above the *x* axes; for *E*–*G*, natural numbers for the reciprocal neutralizing titers also are provided (brackets). Asterisks indicate the significance of differences between the groups. ns, nonsignificant.

To evaluate the replication and immunogenicity of the vaccine candidates in a susceptible animal model, we immunized groups of 30 hamsters intranasally with 5 log_10_ PFU of B/HPIV3/S or B/HPIV3/S-2P candidate vaccines, or B/HPIV3 empty vector (Exp. 1) (Fig. 3*A*). On days 3 and 5 after inoculation, eight hamsters per group were killed to evaluate vector replication in the respiratory tract: nasal turbinates and lungs were harvested from six animals and tissue homogenates were prepared and analyzed by immunoplaque assay (Fig. 3 *B* and *C*), and lungs were harvested from the remaining two animals and analyzed by immunohistochemistry (IHC) (Fig. 3*D*).

As previously reported (19), B/HPIV3 replicated well in nasal turbinates and lungs (6.3 and 5.4 log_10_ PFU/g, respectively, on day 3) (Fig. 3 *B* and *C*), decreasing 10-fold in turbinates by day 5. Day 3 turbinate titers of B/HPIV3/S and B/HPIV3/S-2P were 10- and 100-fold lower, respectively, than empty vector, but surprisingly, increased by day 5, consistent with delayed replication in the upper respiratory tract due to the presence of the insert. Importantly, the mean nasal peak titers of all three viruses independent of the study day were similar.

In the lungs, B/HPIV3 replication remained at a high level over both days (5.4 log_10_ PFU/g) (Fig. 3*C*). Similar to the findings in the nasal turbinates, the mean titers of B/HPIV3/S and B/HPIV3/S-2P (5.0 log_10_ and 4.4 log_10_ PFU/g) were lower than those of the B/HPIV3 empty vector on day 3, although the difference between B/HPIV3 and B/HPIV3/S in the lungs did not reach statistical significance. By day 5, B/HPIV3/S reached about 10-fold higher titers compared to the empty vector on either day, suggesting that the wild-type version of the S protein contributed to vector replication in the lungs. The peak titers of B/HPIV3/S-2P in the lungs were also marginally higher than those of B/HPIV3, but this was not statistically significant.

To determine the stability of S and S2-P protein expression in vivo by the B/HPIV3 vectors, we analyzed lung samples by dual-staining immunoplaque assay; 99.5% and 98.4% of B/HPIV3/S and B/HPIV3/S-2P plaques from lung samples obtained on day 3 after infection stably expressed the S protein, and 99.4% and 97.9% of B/HPIV3/S and B/HPIV3/S-2P plaques obtained on day 5 expressed the S protein (Fig. 3 *C*, *Lower*). Thus, vector expression of both versions of the S protein was stably maintained in vivo. We failed to detect infectious B/HPIV3, B/HPIV3/S, and B/HPIV3/S-2P in homogenized brain, kidney, liver, spleen, or small intestine, showing that the S protein expression did not broaden B/HPIV3 vector tropism in hamsters.

We analyzed antigen expression in the lungs of two immunized animals per group by IHC on days 3 and 5 after immunization; representative IHC images are shown in Fig. 3*D*. We primarily detected B/HPIV3 antigen in columnar epithelial cells lining the bronchioles, as shown in tissue from B/HPIV3, B/HPIV3/S, and B/HPIV3/S-2P–immunized animals obtained on day 5 (Fig. 3 *D*, *Upper*, red arrowheads). SARS-CoV-2 S antigen in animals immunized with B/HPIV3/S and B/HPIV3/S-2P similarly was detected in bronchioles (Fig. 3 *D*, *Lower*, blue arrowheads). Overall, the B/HPIV3 and SARS-CoV-2 S immunostaining patterns did not differ between animals immunized with B/HPIV3/S vs. B/HPIV3/S-2P.

In a small supplementary experiment (Exp. S1) (*SI Appendix*, Fig. S2*A*), we immunized six hamsters per group with B/HPIV3, B/HPIV3/S, or B/HPIV3/S-2P, as described above, to evaluate vaccine replication at a late time point. On day 7, residual B/HPIV3 was detectable only in nasal tissues of a single animal. Residual B/HPIV3/S or B/HPIV3/S-2P was detectable at low titers in some animals in nasal tissues (three and two animals) and in lungs (two and four animals) (*SI Appendix*, Fig. S2 *B* and *C*). Animals in all groups gained weight similarly to mock-immunized control animals from day 0 to day 7 postinoculation, showing that the viruses were well-tolerated in hamsters (*SI Appendix*, Fig. S2*D*).

Taken together, these data demonstrate that following intranasal immunization of hamsters, the B/HPIV3/S and B/HPIV3/S-2P vectors efficiently infected and expressed the SARS-CoV-2 S protein in bronchial epithelial cells, with no obvious difference in tissue distribution between B/HPIV3 expressing the wild-type and the prefusion-stabilized versions of the S protein with ablated S1/S2 cleavage site.

### Serum Antibody Response to Immunization.

We next evaluated serum antibody responses 28 d after intranasal immunization in the remaining animals from Exp. 1 (*n* = 14 animals per group). We measured SARS-CoV-2–neutralizing antibody titers by a 50% neutralizing dose (ND_50_) assay using SARS-CoV-2, strain WA1/2020, a representative of the SARS-CoV-2 lineage A with an S amino acid sequence identical to that expressed by B/HPIV3/S (Fig. 3*E*). As expected, SARS-CoV-2–neutralizing antibodies were not detected in animals immunized with the B/HPIV3 empty vector. B/HPIV3/S induced a very low response of serum SARS-CoV-2–neutralizing antibodies (geometric mean reciprocal ND_50_ titer: 0.86 log_10,_ [1:7.2]), whereas B/HPIV3/S-2P induced ∼12-fold higher titers of SARS-CoV-2–neutralizing antibodies (geometric mean reciprocal ND_50_ titer: 1.95 log_10_ [1:89.1]) (Fig. 3*E*]. We also evaluated the ability of the serum antibodies induced by the B/HPIV3 vectors to neutralize SARS-CoV-2 variants of concern. Sera from immunized hamsters were evaluated in neutralization assays using isolate USA/CA_CDC_5574/2020 of lineage B.1.1.7 (United Kingdom variant, carrying the N501Y, A570D, D614G, P681H, T716I, S982A, and D1118H signature mutations in the S protein), and USA/MD-HP01542/2021 of lineage B.1.351 (South Africa variant, carrying the signature mutations K417N, E484K, N501Y, D614G, and A701V in S) (24, 25).

Remarkably, serum-neutralizing antibody titers induced by B/HPIV3/S-2P to the B.1.1.7 isolate (geometric mean reciprocal ND_50_ titer: 1.97 log_10_ [1:93.3]) (Fig. 3*F*) were comparable to those against WA1/2020 (lineage A). Against the isolate of lineage B.1.351 (Fig. 3*G*), we observed greater animal-to-animal variability in neutralizing titers (geometric mean reciprocal ND_50_ titer: 1.72 log_10_ [1:52.2]). Serum antibodies from B/HPIV3/S-immunized animals only exhibited very low neutralizing activities against these representatives of heterologous lineages, consistent with the low serum neutralizing antibody titers against WA1/2020 (lineage A).

We also measured SARS-CoV-2–specific serum IgG by ELISA using purified preparations of the secreted form of the S-2P protein (Fig. 3*H*) and a fragment (amino acids 319 to 591) of the S protein bearing the RBD as antigens (Fig. 3*I*). Moderate serum IgG titers to the secreted S-2P protein and the RBD were detected in B/HPIV3/S-immunized animals, while significantly stronger IgG responses to the secreted S-2P (13-fold higher) and RBD (10-fold higher) antigens were induced by B/HPIV3/S-2P. We also measured serum IgA titers to S-2P by dissociation-enhanced lanthanide time-resolved fluorescent immunoassay (DELFIA-TRF) (Fig. 3*J*). Both B/HPIV3/S and B/HPIV3/S-2P induced strong S-specific IgA response, and consistent with the IgG response, B/HPIV3/S-2P induced significantly greater IgA titers than B/HPIV3/S (5-fold).

B/HPIV3, B/HPIV3/S, and B/HPIV3/S-2P also induced a strong neutralizing antibody response in a 60% plaque reduction assay against B/HPIV3 (Fig. 3*K*). The antibody response to the B/HPIV3 vector induced by B/HPIV3/S-2P was similar to that induced by the empty B/HPIV3 vector control, while the antibody response induced by B/HPIV3/S was slightly lower than that of the empty vector. These findings support B/HPIV3/S-2P as an excellent candidate vaccine that induces a potent neutralizing antibody response to both SARS-CoV-2 and HPIV3.

### Protection Against Intranasal Challenge with SARS-CoV-2.

**Fig. 4.**
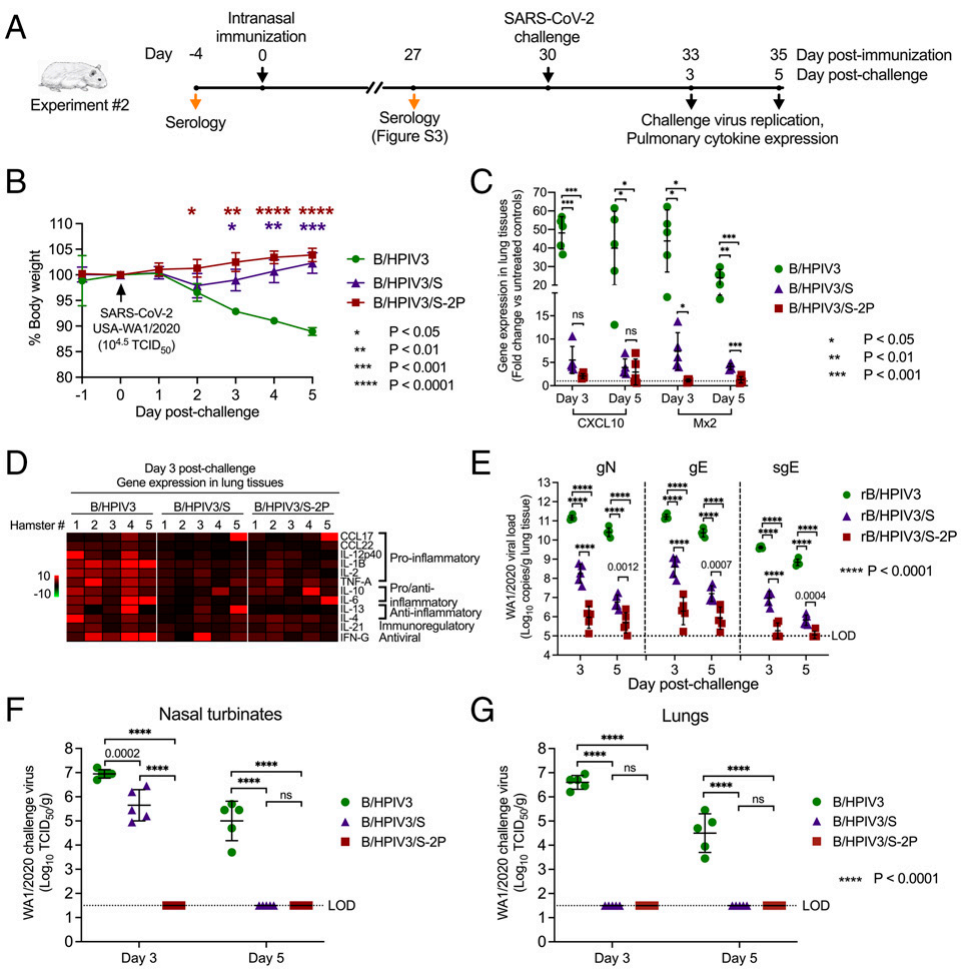
Protection of immunized hamsters against SARS-CoV-2 challenge. (*A*) In Exp. 2, hamsters in groups of 10 were immunized intranasally as described in Fig. 3 and on day 30 postimmunization were challenged intranasally with 4.5 log_10_ TCID_50_ per animal of SARS-CoV-2, strain WA1/2020. (*B*) Weight change after challenge; mean percent and SDs are shown for each group (*n* = 5 animals). Asterisks indicate the significance of differences of B/HPIV3/S and B/HPIV3/S-2P compared to the B/HPIV3-immunized group, determined by two-way ANOVA with Tukey multiple comparison test. (*C* and *D*) Expression of inflammatory cytokines in lung tissues after challenge. On days 3 and 5 postchallenge, five animals per group were killed, and tissues were collected. Total RNA was extracted from lung homogenates. cDNA was synthesized from 350 ng of RNA, and analyzed by qPCR using a custom-made 16-gene hamster-specific Taqman array. qPCR results were analyzed using the comparative threshold cycle (ΔΔC_T_) method, normalized to β-actin. (*C*) Relative gene expression of CXCL10 and Mx2, IFN-inducible antiviral response genes, compared to the mean level of expression of three unimmunized, unchallenged controls (dashed line). (*D*) Heat maps showing expression of 12 immune response genes on day 3 after SARS-CoV-2 challenge, presented as fold-increase (red) or decrease (green) of gene expression over the mean of three unimmunized, unchallenged controls. (*E*) SARS-CoV-2 lung viral loads after challenge, expressed in log_10_ genome copies per gram. To detect viral gN, gE, and sgE of the challenge virus WA1/2020, cDNA was synthesized from total RNA from lung homogenates as described above, and Taqman qPCRs were performed (*n* = 5 animals per time point). (*F* and *G*) SARS-CoV-2 challenge virus titers were determined in homogenates from nasal turbinates (*F*) and lungs (*G*) of five animals per group. (*C*, *E*, *F*, and *G*) Individual titers, means, and SDs are shown for each group. *P* values were determined in two-way ANOVA tests with Tukey multiple comparison tests. LOD, limit of detection; ns, nonsignificant.

To evaluate protection against intranasal SARS-CoV-2 challenge, we immunized hamsters in groups of 10 with B/HPIV3, B/HPIV3/S, and B/HPIV3/S-2P, as described above (Fig. 4*A*) (Exp. 2). The serum antibody response 27 d after immunization (*SI Appendix*, Fig. S3) was comparable to that in Exp. 1 (Fig. 3 *E*, *H*–*J*). We challenged hamsters intranasally on day 30 with 4.5 log_10_ 50% tissue culture infectious dose (TCID_50_) of SARS-CoV-2, isolate WA1/2020 from a preparation subjected to complete-genome deep sequencing to confirm its integrity. Animals were monitored for clinical signs and weight loss (Fig. 4*B*).

During the first 5 d following SARS-CoV-2 challenge, animals immunized with the empty B/HPIV3 vector exhibited moderate weight loss, the sole clinical symptom after challenge (10% average loss by day 5 postchallenge), while animals immunized with B/HPIV3/S and B/HPIV3/S-2P generally continued to gain body weight. The weight loss in the empty B/HPIV3 vector-immunized group reached significant levels compared to the B/HPIV3/S-2P–immunized animals on day 2, and to the B/HPIV3/S-immunized animals on day 3. To evaluate the cytokine response to SARS-CoV-2 3 and 5 d postinfection, we extracted RNA from lung homogenates from five animals on each day and determined the expression of key cytokine genes by Taqman assays (Fig. 4 *C* and *D*). In Fig. 4*C*, results are shown for the two most strongly expressed genes following SARS-CoV-2 challenge in the B/HPIV3 control immunized animals, namely C-X-C motif chemokine ligand 10 (CXCL10), which was about 50-fold and 40-fold increased on days 3 and 5 after challenge relative to unimmunized, unchallenged controls, and myxovirus resistance protein 2 (Mx2), which was about 40- and 20-fold increased on days 3 and 5. CXCL10 is an interferon (IFN)-inducible chemokine and represents a biomarker for the SARS-CoV-2 cytokine storm, providing a correlate of disease severity in COVID-19 patients (26). Mx2 is an IFN inducible antiviral response gene. On days 3 and 5 postchallenge, the protection afforded by immunization with B/HPIV3/S and B/HPIV3/S-2P was evident from the greatly diminished induction of CXCL10 and Mx2, which in the case of B/HPIV3/S-2P was barely detectable, particularly for Mx2 expression.

Extending these results, we evaluated a panel of 12 immune response genes, including proinflammatory cytokines C-C-ligand (CCL)17, CCL22, interleukin (IL)-12p40, IL-1B, IL-2, and tumor necrosis factor-α (TNF-A); immunoregulatory factors IL-10, IL-6, and IL-21; antiinflammatory cytokines IL-13 and IL-4; and IFN-γ (IFN-G) (Fig. 4*D*). Most genes were expressed at a higher level in B/HPIV3 vector control-immunized animals compared to B/HPIV3/S- and B/HPIV3/S-2P–immunized animals on day 3 after challenge, indicating that SARS-CoV-2 challenge induced a strong inflammatory cytokine response in B/HPIV3 empty vector-immunized animals, but not in B/HPIV3/S- and B/HPIV3/S-2P–immunized animals.

We next evaluated challenge virus loads by Taqman assay in lung homogenates using N and E gene-specific assays that detect both SARS-CoV-2 genomic RNA and mRNA, and an assay to specifically detect subgenomic E mRNA (sgE), indicating SARS-CoV-2 mRNA synthesis (Fig. 4*D*). In B/HPIV3 control immunized animals, we detected very high N and E genomic RNA loads, with mean copy numbers of about 11.2 log_10_ and 10.4 log_10_ per gram on days 3 and 5 after challenge, as well as high sgE copy numbers (means of 9.6 and 8.8 log_10_ copies per gram on days 3 and 5). By contrast, in lung homogenates of B/HPIV3/S-immunized animals, means of genomic N and E RNA loads were about 400- to 4,000-fold lower than in B/HPIV3 control-immunized animals on days 3 and 5 after challenge, and mean sgE loads were about 400- and 1,000-fold lower on days 3 and 5 after challenge. In lung homogenates of B/HPIV3/S-2P–immunized animals, the N and E genomic RNA loads were about 4 to 5 log_10_ lower than in B/HPIV3 empty vector-immunized animals, and sgE loads were just above the limit of detection of the assay, with only two (day 3) and one (day 5) of five animals with sgE detectable in lung homogenates.

Finally, we measured SARS-CoV-2 titers in homogenates of lungs and nasal turbinates obtained on days 3 and 5 postchallenge (Fig. 4 *F* and *G*). In nasal turbinates, animals immunized with the empty vector had high mean titers of 7.0 log_10_ and 5.0 log_10_ TCID_50_/g of tissue of challenge SARS-CoV-2 on days 3 and 5, respectively (Fig. 4*F*). In animals immunized with B/HPIV3/S, challenge virus titers in the nasal turbinates were about 20-fold lower on day 3 and undetectable on day 5. Importantly, in animals immunized with B/HPIV3/S-2P, challenge virus was undetectable in nasal turbinates on both days. Animals immunized with B/HPIV3/S or B/HPIV3/S-2P had no detectable virus in lungs on day 3 or day 5, compared with mean titers of 6.6 log_10_ TCID_50_/g and 4.5 log_10_ TCID_50_/g of SARS-CoV-2 in animals immunized with the empty vector (Fig. 4*G*).

Together, our data demonstrate first that B/HPIV3 expressing SARS-CoV-2 S is highly protective against SARS-CoV-2 challenge, and second that the prefusion S protein stabilization substantially enhances immunogenicity and protective efficacy, making this vector an ideal candidate for human clinical trials.

## Discussion

While pediatric SARS-CoV-2 infections are generally mild, they still are associated with a substantial burden of disease, and pediatric SARS-CoV-2 infections contribute to transmission dynamics (27–30). A pediatric vaccine that induces a robust immune response in the upper and lower respiratory tract, in addition to a systemic response, has the potential to strongly restrict SARS-CoV-2 at its primary site of infection and shedding, which should enhance protection and restrict household and community transmissions.

Here we investigated B/HPIV3 as candidate intranasal SARS-CoV-2 vaccine vector. The B/HPIV3 vector is well-tolerated and immunogenic in HPIV3 seronegative young children (17). B/HPIV3 expressing HRSV F protein was similarly well-tolerated in children and infants as young as 2 mo of age (17, 18), and further versions are in clinical development as bivalent HRSV/HPIV3 vaccines (15, 16, 23, 30). Thus, the B/HPIV3 vector is well characterized in previous pediatric clinical studies. We inserted two versions of the SARS-CoV-2 S protein into B/HPIV3: wild-type S protein and a prefusion-stabilized uncleaved form of S.

This work is built on our experience using B/HPIV3 or HPIV3 to express the HRSV F protein (19, 20, 22, 31, 32), which established that: 1) inserting a foreign gene between the N and P genes of B/HPIV3 optimizes insert expression and genetic stability (22); 2) restoring the wild-type assignments at amino acids 263 and 370 in the HPIV3 HN protein (I263T and T370P) removes amino acid substitutions previously inserted as markers and increases the genetic stability of the PIV3 vector backbone (32); and 3) the immunogenicity and protective efficacy of the HRSV F protein is increased modestly by codon optimization, and greatly by stabilizing the F prefusion conformation and modifying the F protein to be packaged efficiently into the B/HPIV3 vector particles (19, 20, 31). In the case of the SARS-CoV-2 S protein, we increased stability in the prefusion conformation, as previously demonstrated by others (9, 11, 33), by including two stabilizing proline mutations that had been effective in stabilizing ectodomains of betacoronavirus S proteins, including those of SARS-CoV-1 and -2 and Middle East respiratory syndrome coronavirus (11, 33). In addition, we ablated the S1/S2 protease cleavage site, as in structural studies of prefusion-stabilized betacoronavirus S proteins (9, 11, 33).

For vaccine development, most prefusion-stabilized versions of the SARS-CoV-2 S protein, including that encoded by the mRNA vaccine (mRNA1273) presently in use, contain two stabilizing 2P mutations (amino acids 986 and 987), the wild-type furin cleavage site, and involve genetic fusion of the ectodomain of the S protein to a C-terminal trimerization motif (9, 10). In contrast, the prefusion-stabilized S protein encoded by B/HPIV3/S-2P contains a 2P stabilized version of the full-length SARS-CoV-2 S protein with ablated S1/S2 furin cleavage site and includes the complete cytoplasmic and transmembrane domains. We chose to ablate the furin cleavage site based on two factors. First, while furin cleavage is not essential for fusion activation of SARS-CoV-2 S, S1/S2 cleavage renders the S2′ cleavage site accessible for TMPRSS2 cleavage, facilitating S2 cleavage and fusion activation (8). Thus, removing the furin cleavage site may optimally stabilize the S protein in the prefusion conformation (13, 34). Second, a version of the S protein with the RRAR cleavage site changed to GSAS did not induce cell–cell fusion, indicating that the multibasic cleavage site is essential for syncytia formation (35); ablation of the furin cleavage site by RRAR-to-GSAS substitutions thus provides for an additional safeguard by rendering the SARS-CoV-2 S protein nonfunctional for mediating fusion in a live viral vector. Importantly, for both B/HPIV3/S and B/HPIV/S-2P, we did not detect any vector replication outside the respiratory tract in the hamster model, indicating that the tropism of the B/HPIV3 vector was unchanged in either case.

To evaluate the effects of prefusion stabilization by 2P mutations and ablating the S1/S2 cleavage site on expression and immunogenicity in the context of full-length S protein, we included B/HPIV3/S as a control, which expressed the unmodified wild-type S protein. When we compared B/HPIV3/S-2P and B/HPIV3/S, we found that in vitro expression of the prefusion-stabilized noncleaved S-2P version was increased. Since antigens had been denatured and reduced before analysis, ablating conformational epitopes, the quantitative differences detected by Western blot should reflect differences in protein expression, rather than differences in antibody reactivity with S-2P compared to S. Thus, increased expression might be due to decreased cellular degradation of conformationally stabilized S, either in the early secretory pathway or in lysosomes, an interesting question for future studies of S protein biogenesis.

Prefusion-stabilization and lack of cleavage were associated with significantly better immunogenicity in the hamster model: compared to B/HPIV3/S, B/HPIV3/S-2P replicated to similar or lower titers in the respiratory tract of hamsters while inducing significantly higher serum IgA and IgG titers to prefusion-stabilized S (5-fold and 13-fold) and the RBD (10-fold), as well as higher (9-fold) titers of SARS-CoV-2–neutralizing serum antibodies to the SARS-CoV-2 isolate WA1/2020, a lineage A representative with an S amino acid sequence identical to that expressed by B/HPIV3/S. Notably, serum antibodies induced by the prefusion stabilized version expressed by B/HPIV3/S-2P neutralized isolates of lineages B.1.1.7 (United Kingdom) and B.1.351 (South Africa), representing two major variants of concern.

Most importantly, following a high-dose intranasal SARS-CoV-2 challenge, we did not detect infectious SARS-CoV-2 challenge virus in respiratory tissues of hamsters immunized with B/HPIV3/S-2P, whereas protection in the upper respiratory tract of animals immunized with B/HPIV3/S was less than complete, at least on day 3 after challenge. Even though the nonstabilized version of the S protein expressed by B/HPIV3/S did not entirely protect the animals from challenge virus infection, it reduced challenge virus replication substantially in magnitude and duration, prevented weight loss and pulmonary induction of inflammatory cytokines in hamsters after challenge, highlighting the overall potency of the B/HPIV3 vector platform.

Unexpectedly, the S-2P version, but not the wild-type S version, was packaged into the B/HPIV3 vector particles. Why prefusion stabilization and ablation of the furin cleavage site resulted in incorporation is not known. In the case of HRSV F protein, the unmodified wild-type protein was not packaged significantly into the vector particle and required substitution of its transmembrane and cytoplasmic tail domains with those of the vector F protein. For HRSV F, packaging into the vector particle resulted in a large increase in the amount and neutralizing capability of serum antibodies induced by immunization, an effect that was similar in quality and magnitude to that of stabilization of HRSV F in the prefusion conformation (19, 31). Similarly, it may be that the packaging of the S-2P protein into the B/HPIV3 particle contributed, in addition to prefusion stabilization, to its greater immunogenicity compared to wild-type S protein.

During the SARS-CoV-1 outbreak of 2002/2003, we developed an experimental B/HPIV3 vaccine expressing the full-length wild-type S protein of SARS-CoV-1 (23, 36) and evaluated it in preclinical studies. Single intranasal administration of the vaccine induced high titers of serum SARS-CoV-1–neutralizing antibodies in hamsters and African green monkeys, and in both models restricted challenge SARS-CoV-1 infection and shedding in the upper and lower respiratory tract (23, 36). In the hamster model, protection against challenge virus replication in the upper respiratory tract was incomplete, but complete in the lungs. In the African green monkey model, we were unable to detect infectious challenge virus in either location, suggesting that intranasal immunization with B/HPIV3 expressing the SARS-CoV-1 S protein conferred complete protection against SARS-CoV-1 challenge. The kinetics of SARS-CoV-2 infection and disease differ from those of SARS-CoV-1: in the case of SARS-CoV-2, infection frequently is followed by an asymptomatic phase in which infected individuals are highly infectious (37, 38). Thus, a vaccine with the ability to induce potent respiratory immunity and restrict shedding from the respiratory tract would be particularly useful for SARS-CoV-2.

Live intranasal viral vaccines in general mimic natural infection and rapidly induce local respiratory tract immunity and systemic immunity following a single vaccine dose. Since it is technically challenging to reliably determine mucosal antibody titers in hamsters, we measured serum IgA titers to the SARS-CoV-2 S protein by a highly sensitive immunoassay, showing that a single intranasal dose of B/HPIV3/S viruses induced a potent IgA response. Live-attenuated and live-vectored vaccines typically induce broad and balanced innate, antibody, and cellular responses, including CD8^+^ and CD4^+^ T cell responses and resident lung T cells (39). In the case of HRSV, because of this broad and balanced response, live-attenuated and live-vectored vaccines are free of the Th2-mediated enhanced disease associated with inactivated HRSV vaccines (40–42). Similarly, replicating SARS coronavirus vaccines are unlikely to prime for the Th2 pulmonary immunopathology that has been seen upon challenge in animal models following immunization with previous inactivated coronavirus vaccine candidates (43–45). A more extensive characterization of the mucosal and systemic immune response following intranasal immunization with B/HPIV3 vectors expressing the SARS-CoV-2 S protein will be included in future studies in rhesus macaques.

The known favorable clinical safety profile of the B/HPIV3 vector will expedite the evaluation of derivatives. The vector is readily manipulated by reverse genetics and has favorable replication and stability characteristics that facilitate manufacture and distribution. A live-attenuated intranasal vaccine is easy to administer and typically is effective in a single dose. Intranasal vector vaccines also are likely to be effective in prime-boost regimens with mRNA vaccines or other vaccines administered by different routes. Based on these features and potential applications and the very promising results in the hamster challenge model, B/HPIV3/S-2P is being advanced to a phase 1 pediatric clinical study, and is expected to be safe and efficacious against both SARS-CoV-2 and HPIV3 in infants and young children. However, in older children and adults, immunity to HPIV3 is prevalent and likely to restrict the replication and immunogenicity of the B/HPIV3 vaccine vector. Similar candidates based on viral vectors that do not commonly infect humans, and thus would be without preexisting immunity in humans, are in development as intranasal vaccines for the nonpediatric population.

## Materials and Methods

### Cells, Viruses, and Reagents.

Human lung epithelial A549 cells (ATCC CCL-185) were grown in F12 medium (ATCC) with 5% FBS. LLC-MK2 rhesus monkey kidney cells, African green monkey Vero cells (ATCC CCL-81), or Vero E6 cells (ATCC CRL-1586) were grown in OptiMEM (Thermo Fisher) with 5% FBS. The Vero E6 cell line is a subclone of Vero cells that provides for efficient replication of SARS-CoV-2. Vero E6 cells were used for SARS-CoV-2 neutralization assays, and to titrate the SARS-CoV-2 challenge virus.

Vero E6 cells stably expressing human TMPRSS2 were generated using the Sleeping Beauty transposase system. The system requires two components, a transposon and a transposase vector (46). To generate the TMPRRS2 transposon, the TMPRRS2 open reading frame was amplified by PCR from Addgene plasmid #53887 [a gift from Roger Reeves, Johns Hopkins University School of Medicine, Baltimore, MD; http://addgene.org/53887; RRID:Addgene_53887 (47)]. PCR primers flanked by unique SfiI restriction recognition sites were used to clone the TMPRRS2 PCR fragment into the Sleeping Beauty transposon plasmid pSBbi-BH (a kind gift from Eric Kowarz, Goethe University, Frankfurt, Germany [Addgene plasmid #60515; http://addgene.org/60515; RRID: Addgene_60515]). pSBbi-BH contains a constitutive bidirectional promoter, flanked by SfiI cloning site for the gene of interest, and by blue fluorescent protein and the hygromycin resistance gene. Following insertion into the SfiI site of pSBbi-BH, the sequence of the TMPRSS2 transposon was confirmed by Sanger sequencing. The transposase plasmid pCMV(CAT)T7-SB100, the second component of the system, was a gift from Zsuzsanna Izsvak, Max Delbrück Center for Molecular Medicine, Berlin, Germany (Addgene plasmid #34879; http://addgene.org/34879; RRID: Addgene_34879). The TMPRSS2 transposon and the transposase plasmid were cotransfected into Vero E6 cells in a molar ratio of 10:1 using TransIT-LT1 Transfection Reagent (Mirus Bio) according to the manufacturer’s instructions. After transfection, Vero E6 cells were grown in the presence of 250 μg/mL hygromycin B Gold (Invivogen) for 2 wk. The integration of the TMPRSS2 transposon was confirmed by flow cytometry detecting blue fluorescent protein expression. TMPRSS2 expression was confirmed by Western blotting using a TMPRSS2 polyclonal antibody (Millipore Sigma, HPA035787). TMPRSS2 expressing Vero E6 cells were further propagated in Dulbecco’s modified Eagle medium with 10% FBS, 1% l-glutamine and 250 µL/mL hygromycin B Gold.

The SARS-CoV-2 USA-WA1/2020 challenge virus (WA1/2020; lineage A; GenBank MN985325 and GISAID: EPI_ISL_404895; obtained from Natalie Thornburg, Sue Gerber, and Sue Tong, Centers for Disease Control and Prevention [CDC], Atlanta, GA) was passaged twice on Vero E6 cells. The amino acid sequence of the S protein of WA1/2020 is identical to that expressed by B/HPIV3/S. The USA/CA_CDC_5574/2020 isolate (lineage B.1.1.7, GISAID: EPI_ISL_751801; sequence deposited by the CDC, isolate obtained from the CDC) and the USA/MD-HP01542/2021 isolate (lineage B.1.351, GISAID: EPI_ISL_890360; sequence deposited by Christopher Paul Morris, Chun Huai Luo, Adannaya Amadi, Matthew Schwartz, Nicholas Gallagher, and Heba H. Mostafa, The Johns Hopkins University, isolate obtained from Andrew Pekosz, The Johns Hopkins University, Baltimore, MD) were passaged on Vero E6 cells stably expressing TMPRSS2. Titration of SARS-CoV-2 was performed by determination of the TCID_50_ in Vero E6 cells (48). Illumina sequence analysis confirmed that the complete genome sequences of the SARS-CoV-2 challenge virus pool and the B.1.1.7 and B.1.351 variants were identical to that of consensus sequences, except for minor backgrounds of reads (<10%). All experiments with SARS-CoV-2 were conducted in Biosafety Level-3 containment laboratories approved for use by the US Department of Agriculture and CDC.

Virus stocks of recombinant B/HPIV3 vectors were propagated on Vero cells at 32 °C and titrated by dual-staining immunoplaque assay, essentially as previously described (20), using a rabbit hyperimmune antiserum against sucrose gradient-purified HPIV3 virions described previously (22), and goat hyperimmune antiserum N25-154 against a recombinantly expressed secreted form (amino acids 1 to 1208) of the SARS-CoV-2 S protein containing two proline substitutions (KV to PP, amino acids 986 and 987) and four amino acid substitutions (RRAR-to-GSAS, amino acids 682 to 685) that stabilize S in the prefusion conformation and ablate the furin cleavage site between S1 and S2 (9). A plasmid encoding this secreted prefusion-stabilized uncleaved S protein (2019-nCoV S-2P_dFurin_F3CH2S) was provided by Barney Graham and Kizzmekia Corbett, Vaccine Research Center, National Institute of Allergy and Infectious Diseases (NIAID), NIH, and Jason McLellan, University of Texas at Austin, Austin, TX. This plasmid was transfected into Expi293 cells and secreted S protein was purified to homogeneity from tissue culture supernatant by affinity chromatography and size-exclusion chromatography and was used to immunize a goat. To perform the dual-staining immunoplaque assay, Vero cell monolayers in 24-well plates were infected with 10-fold serially diluted samples. Infected monolayers were overlaid with culture medium containing 0.8% methylcellulose, and incubated at 32 °C for 6 d, fixed with 80% methanol, and immunostained with the HPIV3-specific rabbit hyperimmune serum to detect B/HPIV3 antigens, and the goat hyperimmune serum to the secreted SARS-CoV-2 S described above to detect coexpression of the S protein, followed by infrared-dye conjugated donkey anti-rabbit IRDye680 IgG and donkey anti-goat IRDye800 IgG secondary antibodies. Plates were scanned with the Odyssey infrared imaging system (LiCor). Fluorescent staining for PIV3 proteins and SARS-CoV-2 S was visualized in green and red, respectively, providing for yellow plaque staining when merged.

### Generation of Recombinant B/HPIV3 Expressing SARS-CoV-2 S Protein.

A cDNA clone encoding the B/HPIV3 antigenome was constructed previously (49) and was previously modified by two amino acid substitutions in the HN protein (I263T and T370P) that removed two sequence markers and restored the fully wild-type sequence (22). The ORF encoding the full-length 1,273 aa wild-type SARS-CoV-2 S protein was codon-optimized for human expression, and a cDNA clone was synthesized commercially (BioBasic). A second version of this cDNA was synthesized that encoded a full-length prefusion-stabilized version of the S protein (called S-2P): this version contained the two proline substitutions and the four amino acid substitutions in the furin cleavage site described above for the secreted S protein (9). The S and S-2P cDNAs were otherwise identical. They were designed to be preceded by a BPIV3 gene junction containing (in 3' to 5' order) a gene-end (AAGTAAGAAAAA), intergenic (CTT), and gene-start (AGGATTAATGGA) motif, followed by a short sequence (CCTGCAGGATG) that contains the initiation ATG (underlined) in a context favorable for translational initiation (Fig. 1) (50). AscI sites were placed flanking each cDNA, and the synthetic DNAs were inserted into a unique AscI site present in the downstream noncoding region of the B/HPIV3 N gene in the B/HPIV3 antigenomic cDNA (Fig. 1). The sequences of the plasmid-borne antigenomic cDNAs were confirmed completely by Sanger sequencing, and plasmids were used to transfect BHK21 cells, clone BSR T7/5, as described previously (23), to produce B/HPIV3/S and B/HPIV3/S-2P recombinant viruses. Virus stocks were grown in Vero cells, and viral genomes purified from recovered virus were sequenced in their entirety by Sanger sequencing from overlapping uncloned RT-PCR fragments, confirming the absence of any adventitious mutations.

### Multicycle Replication of B/HPIV3 Vectors in Cell Culture.

Vero cells in six-well plates were infected in triplicate wells with indicated viruses at an MOI of 0.01 PFU per cell. After virus adsorption, the inoculum was removed, cells were washed, and 3 mL of fresh medium was added to each well followed by incubation at 32 °C for 7 d. At 24 h intervals, 0.5 mL of culture medium was collected and flash-frozen, and 0.5 mL of fresh medium was added to each well. Virus aliquots were titrated together in Vero cells in 24-well plates by the infrared fluorescent dual-staining immunoplaque assay described above.

### SDS/PAGE and Western Blot Analysis.

Vero or A549 cells in 6-well plates were infected with B/HPIV3, B/HPIV3/S, and B/HPIV3/S-2P at an MOI of 1 PFU per cell and incubated at 32 °C for 48 h. Cells were washed once with cold PBS and lysed with 300 µL LDS lysis buffer (Thermo Fisher Scientific) containing NuPAGE reducing reagent (Thermo Fisher Scientific). Cell lysates were passed through a QIAshredder (Qiagen), heated for 10 min at 95 °C, separated on 4 to 12% Bis-Tris NuPAGE gels (Thermo Fisher Scientific) in the presence of antioxidant (Thermo Fisher Scientific), and the resolved proteins were transferred to polyvinylidene difluoride membranes. Membranes were blocked with blocking buffer (LiCor) and incubated with a goat hyperimmune serum to SARS-CoV-2 S, rabbit polyclonal hyperimmune sera against purified HPIV3 (see *Cells, Viruses, and Reagents*, above), rabbit polyclonal hyperimmune sera against an HPIV3 HN peptide (YWKHTNHGKDAGNELETC) (22) or the recombinant purified ectodomain of the HPIV3 F protein (32) in blocking buffer overnight at 4 °C. A mouse monoclonal antibody to GAPDH (Sigma) was included to provide a loading control. Membranes were incubated with infrared dye-labeled secondary antibodies (donkey anti-rabbit IgG IRDye 680, donkey anti-goat IgG IRDye 800 or IRDye 680, and donkey anti-mouse IgG IRDye 800, LiCor). Images were acquired and the intensities of individual protein bands were quantified using Image Studio software (LiCor). The relative abundance of viral proteins was normalized by GAPDH, and presented as fold-change compared to that of the B/HPIV3 vector.

To analyze the protein composition of virus particles, viruses were grown on Vero cells, purified from the supernatant by centrifugation through 30%/60% discontinuous sucrose gradients, and gently pelleted by centrifugation to remove sucrose, as described previously (51). The protein concentration of the purified preparations was determined (Pierce BCA protein assay kit, Thermo Fisher Scientific) prior to the addition of lysis buffer, and 1 µg of protein per lane was used for SDS/PAGE, silver staining (Pierce Silver Staining Kit, Thermo Fisher Scientific), and Western blotting.

### Replication, Immunogenicity, and Protective Efficacy Against SARS-CoV-2 Challenge in Hamsters.

All animal studies were approved by the NIAID Animal Care and Use Committee. In Exp. 1, groups (*n* = 30) of 5- to 6-wk-old female golden Syrian hamsters (Envigo Laboratories), prescreened to be HPIV3-seronegative, were anesthetized and inoculated intranasally with 100 µL of Leibovitz’s L-15 medium (Thermo Fisher Scientific) containing 5 log_10_ PFU of B/HPIV3, B/HPIV3/S, or B/HPIV3/S-2P viruses. On days 3 and 5 postinoculation, six hamsters per group were killed by CO_2_ inhalation, and nasal turbinates, lung, kidney, liver, spleen, intestine, brain, and blood were collected to evaluate virus replication. Lung tissue samples for histology were obtained from two additional hamsters per group on each day. For quantification of B/HPIV3 vector replication, tissues were homogenized in Leibovitz’s L-15 medium, and clarified homogenates were analyzed by dual-staining immunoplaque assay on Vero cells, as described above. On day 28 postimmunization, sera were collected from the remaining 14 animals per group to evaluate the immunogenicity of the vaccine candidates to SARS-CoV-2 and HPIV3. B/HPIV3 vector-specific neutralizing antibodies were detected by a 60% plaque reduction neutralization test (PRNT_60_) (20) on Vero cells in 24-well plates using a version of B/HPIV3 expressing the eGFP protein from the first promoter-proximal genome position. To determine the serum neutralizing antibody response to SARS-CoV-2, 2-fold dilutions of heat-inactivated hamster sera were tested in a microneutralization assay for the presence of antibodies that neutralized the replication of 100 TCID_50_ of SARS-CoV-2 in Vero E6 cells, with four wells per dilution on a 96-well plate. The presence of viral cytopathic effect was read on day 4. The dilution of serum that completely prevented cytopathic effect in 50% of the wells (ND_50_) was calculated by the Reed and Muench formula (52). Serum IgG antibodies to SARS-CoV-2 also were measured by ELISA using two different recombinantly expressed purified forms of S: one was the secreted form of S-2P described above (plasmids generously provided by Barney Graham, Kizzmekia Corbett, and Jason McLellan), and the other was a fragment (amino acids 328 to 531) of the SARS-CoV-2 S protein containing the RBD, obtained from David Veesler through BEI Resources, NIAID, NIH (53). The RBD fragment was expressed from a codon-optimized ORF in Expi293 cells and purified as described above for the secreted S-2P protein. Serum IgA antibodies to the secreted form of S-2P were measured by europium ion-enhanced DELFIA-TRF immunoassay (Perkin-Elmer) following the supplier’s protocol.

In the supplementary Exp. S1, groups (*n* = 6) of 5- to 6-wk-old golden Syrian hamsters were immunized as described above, or mock-immunized with diluent only. Hamsters were weighed daily from day 0 to day 7 postinoculation. On day 7, hamsters were killed by CO_2_ inhalation, and nasal turbinates and lungs were collected to evaluate virus replication.

In Exp. 2, groups (*n* = 10) of 6-wk-old male golden Syrian hamsters were immunized as described above. On day 30 after immunization, hamsters were challenged intranasally with 4.5 log_10_ TCID_50_ of SARS-CoV-2 in 100 µL. Five hamsters per group were killed by CO_2_ inhalation on days 3 and 5 after challenge, and tissues were collected to evaluate challenge virus replication (*n* = 5 per group). The presence of challenge virus in clarified tissue homogenates was evaluated later by TCID_50_ assay on Vero E6 cells.

### qRT-PCR Analysis of Gene Expression in Lung Tissue.

Total RNA was extracted from 0.125 mL of lung homogenates (0.1 g/mL) using the TRIzol Reagent and Phasemaker Tubes Complete System (Thermo Fisher) along with the PureLink RNA Mini Kit (Thermo Fisher) following the manufacturer’s instructions. Total RNA was also extracted from lung homogenates of three control hamsters (nonimmunized and nonchallenged) in the same manner. cDNA was synthesized from 350 ng of RNA by using the High-Capacity RNA-to-cDNA Kit (Thermo Fisher). Low-density Taqman gene arrays (Thermo Fisher) were configured to contain TaqMan primers and probes for 15 hamster (*Mesocricetus auratus*) chemokine and cytokine genes, which were designed based on previous reports (54–56). Hamster β-actin was included as a housekeeping gene. A mixture of cDNA and 2× Fast Advanced Master Mix (Thermo Fisher) was added into each fill port of the array cards for real-time PCR with QuantStudio 7 Pro (Thermo Fisher). qPCR results were analyzed using the comparative threshold cycle (ΔΔC_T_) method, normalized to β-actin, and expressed as fold-changes over the average expression of three uninfected, unchallenged hamsters. Results in Fig. 4*D* are presented as heat maps using the Gene Expression Similarity Investigation Suite (GENESIS program, release 1.8.1, http://genome.tugraz.at).

To detect viral genomic N (gN), E (gE), and sgE mRNA of SARS-CoV-2 challenge virus WA1/2020 in the lung homogenates, cDNA was synthesized from total RNA as described above, and Taqman qPCRs for N, E, and sgE were performed using previously-described primers and probes (57–59) and 2× Fast Advanced Master Mix (Thermo Fisher). Assays were performed on a QuantStudio 7 Pro real-time PCR system (Thermo Fisher). Standard curves were generated using serially diluted pcDNA3.1 plasmids containing gN, gE, or sgE sequences. The sensitivity of the Taqman assay was 10 copies, corresponding to a limit of detection of 5 log_10_ copies per gram of tissue.

### Immunohistopathology Analysis.

Lung tissue samples from hamsters were fixed in 10% neutral buffered formalin, processed through a Leica ASP6025 tissue processor (Leica Biosystems), and embedded in paraffin. Next, 5-μm tissue sections were stained with H&E for routine histopathology. For IHC evaluation, sections were deparaffinized and rehydrated. After epitope retrieval, sections were labeled with goat hyperimmune serum to anti–SARS-CoV-2 S (N25-154) at 1:1,000, and rabbit polyclonal anti-HPIV3 serum (22) at 1:500. Chromogenic staining was carried out on the Bond RX platform (Leica Biosystems) according to manufacturer-supplied protocols. Detection with DAB chromogen was completed using the Bond Polymer Refine Detection kit (Leica Biosystems). The VisUCyte anti-goat HRP polymer (R&D Systems) replaced the standard Leica anti-rabbit HRP polymer from the kit to bind the SARS-CoV-2 S goat antibodies. Slides were finally cleared through gradient alcohol and xylene washes prior to mounting. Sections were examined by a board-certified veterinary pathologist using an Olympus BX51 light microscope and photomicrographs were taken using an Olympus DP73 camera.

### Statistical Analysis.

Datasets were assessed for significance using one-way ANOVA with Tukey’s multiple comparison test using Prism 8 (GraphPad Software). Data were only considered significant at *P* ≤ 0.05.

## Supplementary Material

Supplementary File

## Data Availability

All study data are included in the main text and *SI Appendix*.
