# Supplementary material for: A single intranasal dose of a live-attenuated parainfluenza virus-vectored SARS-CoV-2 vaccine is protective in hamsters
Source: Proc Natl Acad Sci U S A. 2021 Dec 7;118(50):e2109744118. doi: 10.1073/pnas.2109744118 (PMC8685679; doi:10.1073/pnas.2109744118)
Supplement: Supplementary File [file pnas.2109744118.sapp.pdf]

PNAS
www.pnas.org

Supplementary Information for

A single intranasal dose of a live-attenuated parainfluenza virus-vectored SARS-CoV-2 vaccine is protective in hamsters

Xueqiao Liu[a1], Cindy Luongo[a1], Yumiko Matsuoka[a1], Hong-Su Park[a], Celia Santos[a], Lijuan Yang[a], Ian N. Moore[b], Sharmin Afroz[a], Reed F. Johnson[c], Bernard A. P. Lafont[c], Craig Martens[d], Sonja M. Best[e], Vincent J. Munster[e], Jaroslav Hollý[f], Jonathan W. Yewdell[f], Cyril Le Nouën[a2*], Shirin Munir[a2*], Ursula J. Buchholz[a2*]

[a] RNA Viruses Section, Laboratory of Infectious Diseases, National Institute of Allergy and Infectious Diseases, National Institutes of Health, Bethesda, MD 20892, USA;
[b] Infectious Disease and Pathogenesis Section, Comparative Medicine Branch, National Institute of Allergy and Infectious Diseases, National Institutes of Health, Bethesda, MD 20892, USA;
[c] SARS-CoV-2 Virology Core, Laboratory of Viral Diseases, National Institute of Allergy and Infectious Diseases, National Institutes of Health, Bethesda, MD 20892, USA;
[d] Research Technologies Section, Rocky Mountain Laboratories, National Institute of Allergy and Infectious Diseases, National Institutes of Health, Hamilton, MT 59840, USA;
[e] Laboratory of Virology, Rocky Mountain Laboratories, National Institute of Allergy and Infectious Diseases, National Institutes of Health, Hamilton, MT 59840, USA;
[f] Cellular Biology Section, Laboratory of Viral Diseases, National Institute of Allergy and Infectious Diseases, National Institutes of Health, Bethesda, MD 20892, USA.

[1] X.L., C.L. and Y.M. contributed equally to this work
[2] C.L.N., S.M. and U.J.B. contributed equally to this work

Corresponding Authors.
Cyril Le Nouen, Email: lenouenc@niaid.nih.gov
Shirin Munir, Email: munirs@niaid.nih.gov
Ursula Buchholz, Email: ubuchholz@niaid.nih.gov

This PDF file includes:

Figures S1, S2, S3

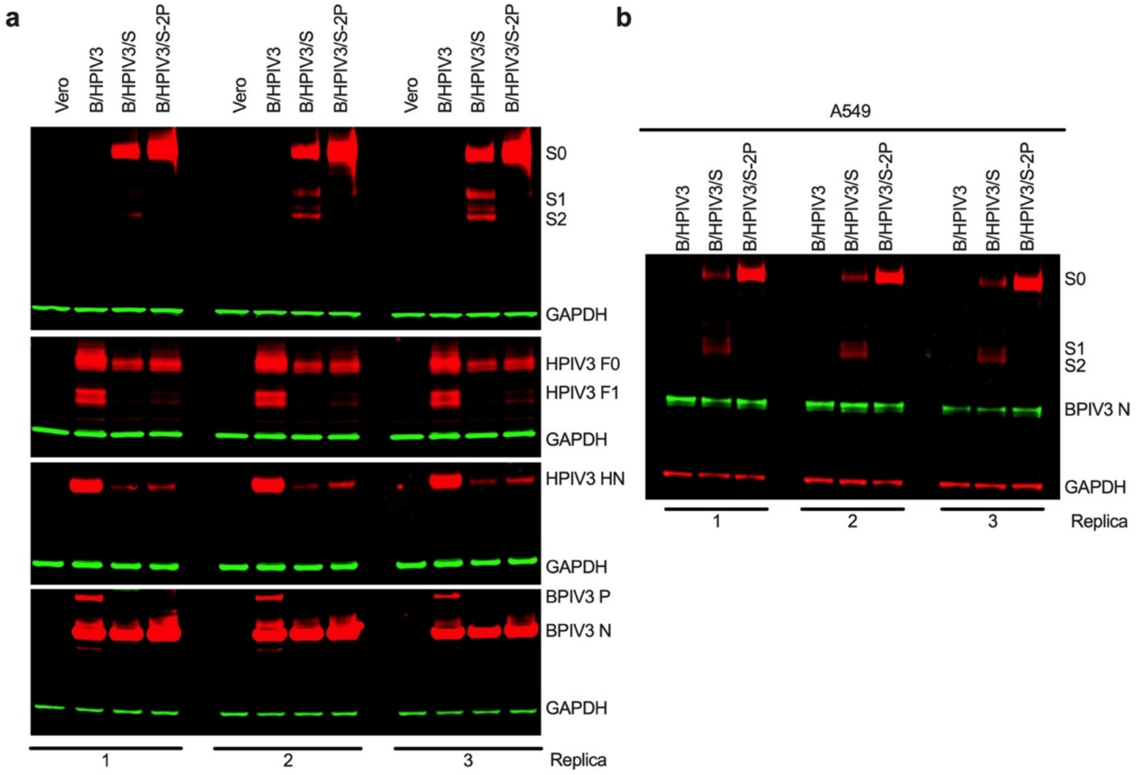

Figure S1. Expression of the BPIV3 N and P proteins, the HPIV3 HN and F proteins, and the SARS-CoV-2 S protein in Vero and A549 cells infected with the indicated viruses. As described for the experiment shown in Figure 2, Vero (**a**) and A549 cells (**b**) were infected with the indicated viruses at an MOI of 1 PFU/cell, and cell lysates were prepared 48 h post-infection, separated by gel electrophoresis under denaturing and reducing conditions and subjected to Western blot analysis. GAPDH was included as loading control. (**a**) The experiment in Vero cells was performed three times, and samples were analyzed side-by-side in Western blots. The expression of each protein was normalized to GAPDH, and protein expression was compared to that of B/HPIV3 from the same experiment. In case of the SARS-CoV-2 S protein, expression by B/HPIV3/S-2P was compared to B/HPIV3/S from the same experiment. The relative levels of expression, determined in 3 independent experiments, are shown in Figure 2b

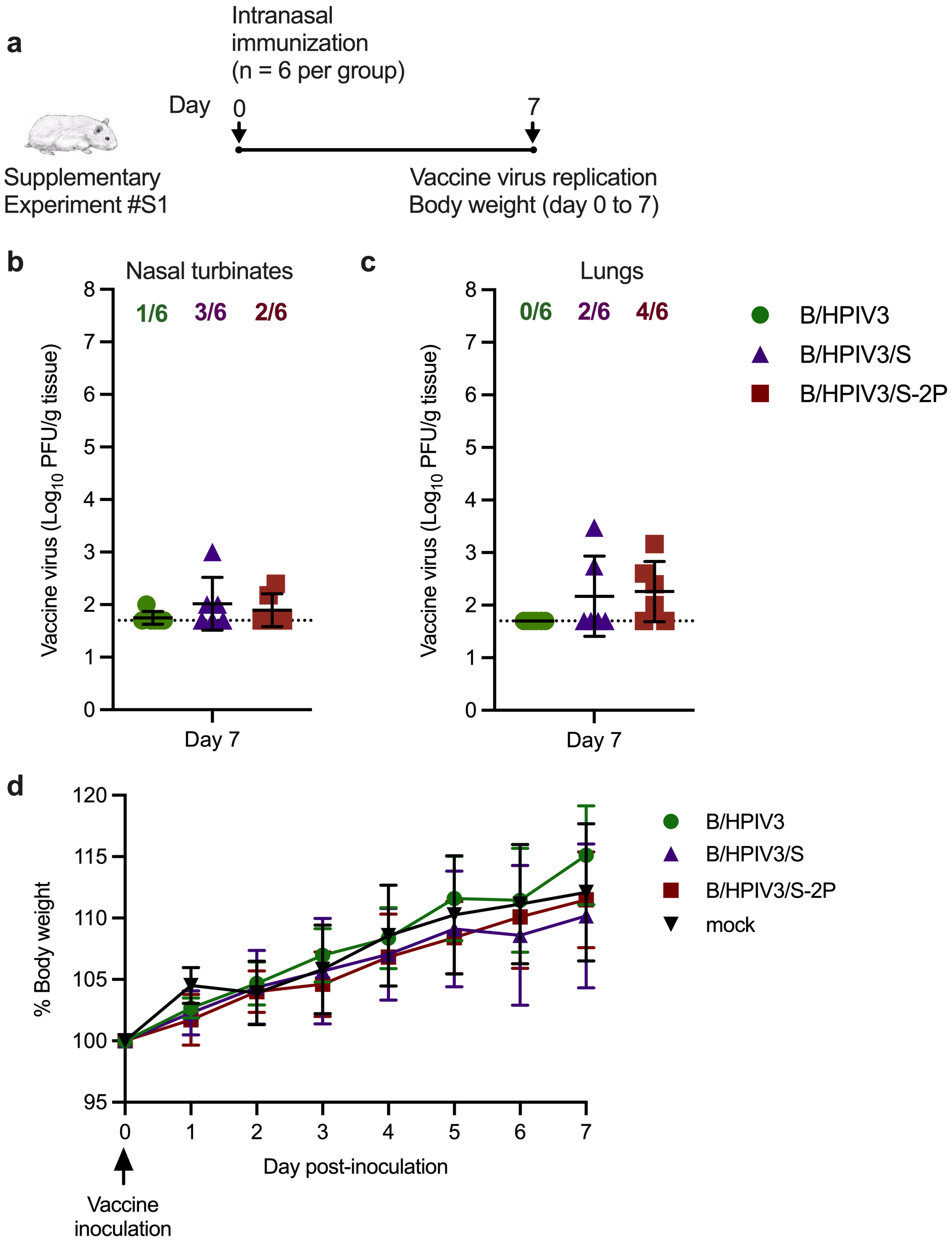

Figure S2. Replication of B/HPIV3 viruses in hamsters on day 7 post-inoculation, and weight change after immunization (a) In Supplementary Experiment #S1, Hamsters in groups of six were mock-immunized or immunized intranasally with 5 $\log_{10}$ PFU of the indicated viruses as described in Figure 3. **(b, c)** On day 7, animals were sacrificed and the viral titers in the nasal turbinates **(b)** and lungs **(c)** were determined by dual-staining immunoplaque assay; mean and SD are shown for each group. The number of hamsters per group with replicating virus is indicated at the top of each graph. The limit of detection is indicated by a dotted line. (**d**) Hamsters were weighed daily from day 0 to day 7 post inoculation, and the weight change was calculated as a percentage of the weight at day 0; mean percent and standard deviations (SD) are shown for each group (n = 6 animals).

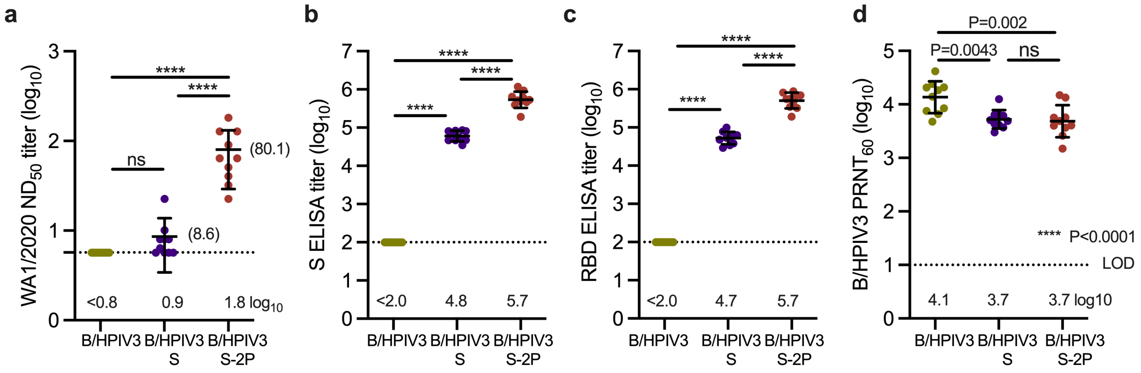

Figure S3. Immunogenicity of B/HPIV3 vectors (Experiment #2). In Experiment #2 (Figure 4(a)), six-week-old golden Syrian hamsters (n=10 animals per group) were inoculated intranasally with 5 $\log_{10}$ PFU of the indicated viruses, following the same procedures as in Experiment #1. (**a, b, c**) Sera were collected on day 27 and serum antibody titers were evaluated to determine the 50% SARS-CoV-2 neutralizing titers (ND_{50}) (**a**), or the IgG ELISA titers to the S protein (**b**) or the RBD (**c**). (**d**) The 60% plaque reduction neutralization titers ($PRNT_{60}$) to B/HPIV3 were also evaluated. Mean $\log_{10}$ antibody titers are indicated below the dotted line; natural numbers for the reciprocal neutralizing titers are also provided (a, in brackets). LOD, limit of detection, indicated by dotted lines. Asterisks indicate the significance of differences between the groups. ns, non-significant.
